# Delivery of systemic anti-cancer therapy during the COVID-19 pandemic

**DOI:** 10.1007/s11845-021-02631-1

**Published:** 2021-05-12

**Authors:** Orla Fitzpatrick, Roisin Ní Dhonaill, Anna Linehan, Zac Coyne, Maeve Hennessy, Maeve Clarke, Elizabeth McGee, Fiona Barrett, Deborah O’Doherty, Carla Matassa, Teresa Doyle, Allyson Christie, Bryan Hennessy, Liam Grogan, Patrick G. Morris, Oscar S. Breathnach, Darren Cowzer

**Affiliations:** 1 Medical Oncology Department, Beaumont Hospital, Dublin, Ireland; 2 Cancer Clinical Trials and Research Unit, Beaumont Hospital, Dublin, Ireland

**Keywords:** COVID-19, Oncology, Screening, Treatment

## Abstract

**Background:**

The first confirmed case of COVID-19 in Ireland was on February 29th 2020. From March until late April, the number of cases increased exponentially. The delivery of anti-cancer therapy during the COVID-19 pandemic was extremely challenging. In order to balance the benefits of continuing anti-cancer therapy with the associated increased hospital visits, combined with the risk of COVID-19 infection, we undertook a series of system changes in the delivery of cancer care.

**Methods:**

Patients who attended our dayward over a 4-month period were included. Data were obtained from patient and chemotherapy prescribing records. Patients were screened for symptoms of COVID-19 at two separate timepoints: prior to their visit via telephone, and using a symptom questionnaire on arrival at the hospital. If patients displayed COVID-19 symptoms, they were isolated and a viral swab arranged.

**Results:**

A total of 456 patients attended from January 1st to April 30th. The numbers of visits from January to April were 601, 586, 575, and 607, respectively. During this period, there were 2369 patient visits to the dayward and 1953 (82%) intravenous regimens administered. Of the 416 visits that did not lead to treatment, 114 (27%) were scheduled non-treatment review visits, 194 (47%) treatments were held due to disease-related illness, and 108 (26%) treatments were held due to treatment-related complications.

Screening measurements were implemented on March 18th due to rising COVID-19 prevalence in the general population. Overall, 53 treatments were held due to the screening process: 19 patients (36%) elicited COVID-19 symptoms via telephone screening; 34 patients (64%) were symptomatic in our pre-assessment area and referred for swabs, of which 4 were positive. Those with a negative swab were rescheduled for chemotherapy the following week.

**Conclusions:**

With careful systematic changes, safe and continued delivery of systemic anti-cancer therapy during the COVID-19 pandemic is possible.

## Introduction

The delivery of systemic anti-cancer therapy during the COVID-19 pandemic has been challenging. Increased hospital visits and active anti-cancer therapy have been described as potential risk factors for developing more severe infection [1]. There is an ever-growing body of evidence reporting on the outcomes of COVID-19 infection in early versus advanced malignancies, with haematological and lung malignancies being associated with more severe infection and a higher death rate [2–5]. Globally, it is a concerning time for immunosuppressed patients, especially in the case where treatments can both prolong life from a cancer perspective, yet potentially increase the risk of a poor outcome with COVID-19 infection [6].

The American Society of Clinical Oncology (ASCO) and the European Society of Medical Oncology (ESMO) have advised the prioritisation of patients with life threatening or clinically unstable disease, and the consideration of treatment delays in those where there is a limited magnitude of benefit [7]. The potential risks of delaying and omitting anti-cancer treatments are a major concern. The risk of progression of disease due to delayed treatments, along with the decreased ability to monitor and manage the symptom burden in patients due to reduced patient contact, is a challenging issue. Recent studies have highlighted the emotional impact of having treatments held, with patients expressing their concerns about vulnerability for disease progression or recurrence [8].

Although the role for telemedicine and virtual patient reviews has expanded exponentially during this pandemic and remains an option to decrease the number of patient visits for those on follow-up or surveillance in the outpatient clinic setting, it does have significant limitations [9, 10]. Telemedicine does not allow for clinicians to examine and fully assess patients prior to prescribing systemic anti-cancer therapies [9]. In order to balance the benefits of continuing systemic anti-cancer therapy with the undoubted risks associated with COVID-19, we undertook a series of system changes in the delivery of cancer care at our oncology centre. Our aim was to determine whether these system changes would allow systemic anti-cancer treatments to proceed as planned according to well established protocols, as well as attempting to limit patients from contracting COVID-19 infection at our healthcare facility.

## Methods

### Setting

This research was carried out in Beaumont Hospital medical oncology department in Dublin, Ireland, specifically focusing on the oncology dayward where outpatients attend to receive intravenous systemic anti-cancer therapy.

### Patients

Patients who attended our oncology dayward over a 4-month period were included. All patients receiving intravenous systemic anti-cancer therapy were included, regardless of cancer type or stage. Patients receiving oral chemotherapy were reviewed at a different location and therefore were excluded.

### System changes

Prior to the pandemic, patients entered the dayward via the main hospital entrance, without any prescreening. From March 18th, patients were screened for symptoms of COVID-19 infection at two separate timepoints: firstly, the day prior to their visit via telephone to assess symptoms and to ascertain any close contacts with confirmed or suspected cases and, secondly, using a symptom questionnaire in combination with temperature checks in a pre-assessment area on arrival at the hospital. This pre-assessment area was established using a unique hospital entrance solely for oncology patients, in order that they did not have to transit through the main hospital thereby limiting contact with other patients, visitors, and staff. Patients arrived at this area at staggered time points to allow social distancing to be maintained, and they were provided with a facemask prior to transiting to the day oncology ward where treatments are given.

If patients displayed COVID-19 symptoms, they were isolated, and a viral swab was arranged for the same day. Overall, attendance at our centre was reviewed prior to the pandemic, and post-initiation of these screening tools. These system changes to the delivery of treatment were implemented on March 18th, within 24 h of the national quarantine measures being announced in Ireland.

### Data collection

Data were obtained from electronic patient records and chemotherapy prescribing records.

## Results

**Fig. 1:**
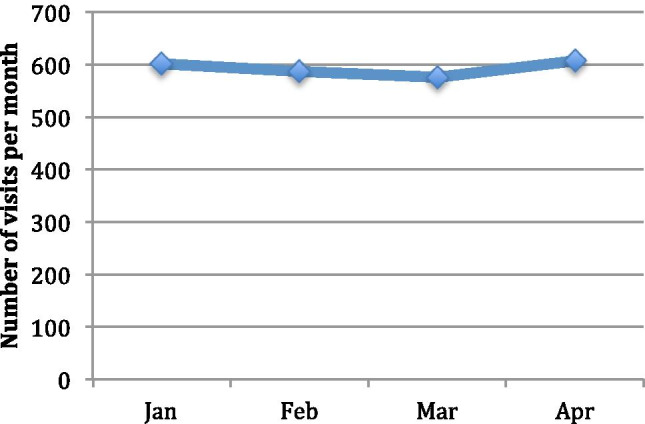
The total number of patient visits per month attending the day oncology ward for systemic anti-cancer therapy during the COVID-19 pandemic

A total of 456 patients attended from January 1st to April 30th (Table 1). During this time, there were 2369 patient visits to the oncology dayward and 1953 intravenous treatments administered. The number of visits per month to our centre remained consistent from January to April with 601, 586, 575, and 607 visits, respectively (Fig. 1). Four hundred sixteen (18%) visits did not lead to systemic treatments, 114 (27%) of which were scheduled non-treatment visits. These scheduled non-treatment visits included the following: treatment education and consent, scheduled imaging, receiving imaging results, insertion of long-term central venous access, and repeat blood tests. A further 194 (47%) treatments were held due to disease-related illness, and 108 (26%) treatments were held due to treatment-related complications.

**Table 1:** Patient demographics of the 456 patients attending the day oncology ward

Total number of patients	456
Age (years)	
Median	59
Range	23–86
Gender	
Male	49% (225)
Female	51% (231)
Cancer type	
Lower GI	21% (96)
Breast	20.4% (93)
Upper GI	14.7% (67)
Brain	13.8% (63)
Lung	11.8% (54)
Melanoma	4.4% (20)
Head and neck	4.4% (20)
Prostate	2.4% (11)
Bladder/renal	2.2% (10)
Other	2% (9)
Ovarian	1.8% (8)
Testicular	1.1% (5)

Telephone screening identified 19 patients as having COVID-19 symptoms or contacts with a suspected or confirmed case. Thirty-four patients were symptomatic on arrival at our pre-assessment area. Of these 53 patients referred for swabs, 4 tested positive, and results were available in less than 24 h of testing. Those with a negative swab were rescheduled for chemotherapy the following week.

## Discussion

COVID-19 has highlighted multiple areas of concern for the delivery of systemic anti-cancer therapy during a pandemic. We have successfully shown that instituting strict screening procedures can allow systemic anti-cancer treatment to be delivered as planned. These system changes allowed us to continue to review patients in person, in a safe setting for both patients and healthcare staff. In our centre, the number of patient visits per month remained stable throughout the first wave of the COVID-19 pandemic. Although some patients were concerned about the risk of COVID-19, many were concerned about stopping or holding treatments during this time, and the associated risk of progression of their cancer [11]. Continuing to provide treatments was essential for many patients, both psychologically and from an oncology perspective.

Although there have been contrasting studies regarding outcomes of COVID-19 infection in cancer patients [5, 12], the safety of patients, including limiting the number of patient contacts, remains important. Developing screening processes appears critical to minimise the chance of COVID-19 transmission within our dayward, with this importance being extended to any clinical area experiencing a high volume of immunosuppressed patients [13–15].

Access to undergo same-day COVID-19 testing enhanced our screening process if there were concerns for infection, thereby avoiding prolonged wait times for testing in the community. Initially, wait times in the community were up to 1 week to be tested, and up to 10 days to be resulted due to testing backlogs. Eventually, national testing capacity was increased, but wait times for results in the community remained between 48 and 72 h. Bypassing these wait times allowed treatments to be quickly rescheduled and allowed most anti-cancer treatments to continue as per their regimen.

COVID-19 has impacted many patients psychologically, as well as affecting treatment delivery. The system changes implemented here prevented patients from bringing family members to their appointments in order to limit the numbers of people attending the dayward. There is a large psychological impact of social isolation on high-risk patients, and it is yet unclear as to whether receiving systemic treatment alone versus with a family member or friend has the capacity to worsen this impact [16, 17].

The data about COVID-19 infection in patients on systemic anti-cancer treatment are evolving in line with the pandemic. Both active haematological and lung malignancies, as well as lymphopaenia, are associated with worse outcomes, but there is a growing database that recent cytotoxic chemotherapy is not [5, 18]. Receiving systemic anti-cancer treatment from 14 to 90 days prior to COVID-19 infection did not increase ICU admission rates or death rates [5]. This emphasizes the importance of maintaining chemotherapy protocols in a safe environment as many countries plan for a second wave of COVID-19 outbreaks.

As the national guidelines about quarantine and preventing the spread of COVID-19 infection begin to change in Ireland and internationally, it is important to continue these measures as the number of social contacts that patients have will likely increase dramatically. The changes made within our institution were quickly instituted and easily maintained and allowed systemic anti-cancer treatments to be delivered on time, thereby attempting to protect vulnerable patients [19].
